# Draft Genome Sequence of Paraclostridium bifermentans subsp. *muricolitidis* Strain PAGU 1678^T^, Which Exacerbates Pathosis in a Mouse Model of Ulcerative Colitis

**DOI:** 10.1128/MRA.00780-21

**Published:** 2021-09-16

**Authors:** Ryo Kutsuna, Tohru Miyoshi-Akiyama, Junko Tomida, Yoshiaki Kawamura

**Affiliations:** a Department of Microbiology, School of Pharmacy, Aichi Gakuin University, Nagoya, Aichi, Japan; b Pathogenic Microbe Laboratory, Research Institute, National Center for Global Health and Medicine, Shinjuku, Tokyo, Japan; University of Rochester School of Medicine and Dentistry

## Abstract

Paraclostridium bifermentans subsp. *muricolitidis* strain PAGU 1678^T^ was isolated from rat feces and has the ability to exacerbate pathosis in a mouse model of ulcerative colitis. Here, we report the draft genome sequence of the 3,471,060-bp chromosome of strain PAGU 1678^T^.

## ANNOUNCEMENT

Ulcerative colitis (UC) is classified as an inflammatory bowel disease, together with Crohn’s disease. The main symptoms of UC are bloody diarrhea and abdominal pain (1). The number of UC patients has been increasing yearly worldwide, but the specific cause of UC is unknown (2). Currently, UC is considered to be caused by multiple factors, such as genetic susceptibility, environmental factors, immune dysregulation, and microbial flora (3). Using a mouse model of UC (4, 5), we found that strain PAGU 1678^T^ was significantly increased in the feces and had the ability to exacerbate colitis by experimental infection (6). Later taxonomic studies revealed that strain PAGU 1678^T^ was a novel subspecies of Paraclostridium bifermentans (7). Here, we report the draft genome sequence of Paraclostridium bifermentans subsp. *muricolitidis* strain PAGU 1678^T^.

Strain PAGU 1678^T^ was incubated at 37°C for 24 h under anaerobic conditions (10% CO_2_, 10% H_2_, 80% N_2_) on GAM agar plates (Nissui) (6). Genomic DNA was extracted and purified from a single colony using a Wizard genomic DNA purification kit (Promega). DNA libraries were prepared using a Nextera XT library preparation kit (Illumina) according to the manufacturer’s instructions. The genome sequence was determined using 2 × 150-bp paired-end reads (14,989,552 reads) obtained with an Illumina HiSeq X Ten sequencer. The final coverage of the genome was 1,061×. These reads were trimmed on the basis of base quality (quality score limit of 0.05) and removed if the reads had more than two ambiguous nucleotides or they were less than 15 bp in length, using CLC Genomics Workbench v9.5.2 (CLC bio). The software was operated using default settings and parameters unless otherwise specified. Automated annotation was carried out with the DDBJ Fast Annotation and Submission Tool (https://dfast.nig.ac.jp) with default parameters.

Strain PAGU 1678^T^ was assembled into 126 contigs (*N*_50_ = 82,995 bp) and had a genome length of 3,471,060 bp (G+C content of 28.1%). The chromosome contained 3,398 predicted protein-coding sequences, 7 rRNAs, and 70 tRNAs. BLAST calculations were performed using GENETYX NGS v15 to calculate the average nucleotide identity (ANI) between strain PAGU 1678^T^ and Paraclostridium bifermentans JCM 1386^T^.

The ANI homology value of 96% suggested that strain PAGU 1678^T^ is the same species as P. bifermentans. The genomic sequence information for this strain provides important data that may lead to elucidation of the mechanism underlying the development of UC.

### Data availability.

The whole-genome sequence of P. bifermentans subsp. *muricolitidis* PAGU 1678^T^ and the raw sequence data have been deposited in DDBJ/EMBL/GenBank under the accession numbers BOQA01000001 to BOQA01000126 and DRA007030, respectively.

## References

[1] Kornbluth A, Sachar DB, Practice Parameters Committee of the American College of Gastroenterology. 2010. Ulcerative colitis practice guidelines in adults: American College of Gastroenterology, Practice Parameters Committee. Am J Gastroenterol 105:501–523. doi:10.1038/ajg.2009.727. 20068560

[2] Matsuoka K, Kobayashi T, Ueno F, Matsui T, Hirai F, Inoue N, Kato J, Kobayashi K, Kobayashi K, Koganei K, Kunisaki R, Motoya S, Nagahori M, Nakase H, Omata F, Saruta M, Watanabe T, Tanaka T, Kanai T, Noguchi Y, Takahashi KI, Watanabe K, Hibi T, Suzuki Y, Watanabe M, Sugano K, Shimosegawa T. 2018. Evidence-based clinical practice guidelines for inflammatory bowel disease. J Gastroenterol 53:305–353. doi:10.1007/s00535-018-1439-1. 29429045 PMC5847182

[3] Choi CH, Moon W, Kim YS, Kim ES, Lee BI, Jung Y, Yoon YS, Lee H, Park DI, Han DS, IBD Study Group of the Korean Association for the Study of Intestinal Diseases. 2017. Second Korean guidelines for the management of ulcerative colitis. Intest Res 15:7–37. doi:10.5217/ir.2017.15.1.7. 28239313 PMC5323310

[4] Low D, Nguyen DD, Mizoguchi E. 2013. Animal models of ulcerative colitis and their application in drug research. Drug Des Devel Ther 7:1341–1357. doi:10.2147/DDDT.S40107. PMC3829622 24250223

[5] Whittem CG, Williams AD, Williams CS. 2010. Murine colitis modeling using dextran sulfate sodium (DSS). J Vis Exp 35:1652. doi:10.3791/1652. PMC2841571 20087313

[6] Kutsuna R, Tomida J, Morita Y, Kawamura Y. 2018. *Paraclostridium bifermentans* exacerbates pathosis in a mouse model of ulcerative colitis. PLoS One 13:e0197668. doi:10.1371/journal.pone.0197668. 29782507 PMC5962066

[7] Kutsuna R, Miyoshi-Akiyama T, Mori K, Hayashi M, Tomida J, Morita Y, Tanaka K, Kawamura Y. 2019. Description of *Paraclostridium bifermentans* subsp. *muricolitidis* subsp. nov., emended description of *Paraclostridium bifermentans* (Sasi Jyothsna et al., 2016), and creation of *Paraclostridium bifermentans* subsp. *bifermentans* subsp. nov. Microbiol Immunol 63:1–10. doi:10.1111/1348-0421.12663. 30549099

